# Abstract of Clinical Lecture in Medical Wards of Mercy Hospital, on Cases of Erysipelas and Diphtheria

**Published:** 1881-03

**Authors:** N. S. Davis

**Affiliations:** Professor of Practical and Clinical Medicine in Chicago Medical College


### ARTICLE III.

ABSTRACT OF A CLINICAL LECTURE IN THE MEDICAL WARDS OF THE MERCY HOSPITAL, FEB. 11, 1881, ON CASES OF ERYSIPELAS AND DIPHTHERIA. By N. S. Davis, Professor of Practical and Clinical Medicine in Chicago Medical College.

GENTLEMEN:—The first case to which I invite your attention is the same that you examined in Ward Number 36, two weeks since. The patient is a woman, aged about 30 years, usually enjoying good health. Three days prior to your first examination she had been attacked with sporadic erysipelas of the face, commencing with general fever and a red spot on the ridge of the right side of the face near the wing of the nose.

You will remember that when you saw her, three days after the initial symptoms, the erysipelatous inflammation had extended over the whole face, except the chin, causing sufficient tumefaction to close the eyes, and give the whole face, together with the right ear, a swollen and intensely red appearance. The pulse was 110 per minute; respiration a little accelerated; temperature 103° F.; skin dry; tongue lightly covered with a white fur; urine scanty and highly colored; bowels inactive, and stomach so irritable as to dispose her to reject both drink and medicine. I then pointed out to you the diagnostic features of the disease, its supposed dependence upon a specific organic poison, and the remedies that experience has demonstrated to be of most value in its treatment; and I call your attention to it now, solely to enable you to know the subsequent progress of the case. The patient had been admitted into the ward two days before your previous visit, and had been given tincture of chloride of iron in doses of 1.500 grams every three hours, well diluted with water, and the inflamed surface covered with cloths wet in carbolated water.

The chief object of the latter was to mitigate the unpleasant heat of the surface, outward applications exerting little or no control over the progress of the disease, while the preparation of iron given internally was relied upon to correct the morbid condition of the blood and shorten the duration of the disease. But you will remember that the patient had not been able to retain the iron in the stomach, and in consequence, I told you we would substitute in its place the sulphite or hypo-sulphite of soda, which I had found by clinical experience to have some influence over the progress of this disease. It was accordingly given in doses of 66 centigrams, dissolved in mint water, repeated every two hours. This was continued three days, during which the inflammation continued to extend, occupying the whole head, face, and neck down upon the right shoulder. Although the disease thus continued actively to spread on the surface, the swelling of the face and eye-lids had begun to diminish; the stomach was retentive; the temperature was 101° F., and the tumefaction of the new parts attacked was slight, and presented but few vesications. But she complained of feeling very weak. The interval between the doses of the sulphite of soda was lengthened to four hours, and one gram of tincture of iron, with 13 centigrams of quinine given between, with milk and beef tea for nourishment. The inflammation continued to spread superficially down the posterior surface of the body to near the hips, where it ceased and has since rapidly disappeared, the patient being now fully convalescent. You will perceive from the history just given that in this case the duration of the disease was just two weeks, and that it extended over a greater amount of surface than usual. Yet the intensity of the symptoms was much lessened from the end of the first week to the time of their disappearance.

DIPHTHERIA.—The next patient to whom I call your attention is this man, who was admitted into the Hospital one week since, having commenced feeling sick two or three days previously.

At the time of admission he complained of headache and severe sore throat. His pulse was 100 per minute; temperature 102° F.; skin dry; tongue coated; the whole arch of the fauces intensely red, tumefied, with a large, irregular ulcer on each tonsil, and two or three small spots of white membranous exudation on the margin of the ulcer on the left tonsil. Two or three of the lympathic glands on the sides of the neck below the tonsils were swollen and tender to pressure, the voice hoarse, and deglutition difficult. These symptoms seemed to indicate clearly enough a case of severe diphtheria with only slight membranous exudation, and an unusually early establishment of ulceration and general muscular weakness.

*Treatment.*—He was directed to take 1.5 grams of tincture of chloride of iron well diluted with sweetened water every two hours for twenty-four hours, and then every three hours thereafter, and to gargle the throat every three hours with a solution of benzoate of soda (8 grams to 200 grams of water). He was also directed to take 13 centigrams of sulph. quinine three times a day, and milk, with farinaceous articles for nourishment. This treatment was continued without change until yesterday, when finding the patient still having a moderate general fever, with continuous severe headache, and only a slow improvement in the appearance of the throat, the iron and quinine were omitted, under the impression that they might be increasing the headache, and in their place was ordered 66 centigrams of bromide with 33 centigrams of iodide of potassium every four hours. To-day he has less headache and some less fever; yet if you examine his throat you will see that the uvula and whole arch of the fauces, with the tonsils, are still red and tumefied, with large, irregular ulcers on the face of each tonsil, but no white membranous exudation and no fœtid odor. The hoarseness and difficulty of swallowing are less than when first admitted, and the ulcers on the tonsils not so deep, but one or two glands on each side of the neck, beneath the angle of the jaw, are still slightly swollen. (The patient being placed in an easy position, with a favorable light, all the members of the Clinical class present were enabled to make personal examination of the throat.)

It is hardly necessary to remind you that cases of disease included under the name of diphtheria differ widely from each other in their degree of severity and in some of their more prominent symptoms. Some practitioners have fallen into the habit of calling almost every case of simple catarrhal inflammation of the fauces and tonsils, diphtheria. Others call no case diphtheria, unless it presents well marked membranous exudation on some part of the inflamed surface. The latter is, of course, much more nearly correct; and yet in almost all seasons, when diphtheria is prevailing with more than ordinary severity, a few cases will be met with characterized by the usual general febrile condition, a rapidly developed dark red and much tumefied condition of the fauces and tonsils, painful deglutition, and slight enlargement of some of the lymphatic glands below the angle of the jaw; but without any perceptible white exudation, or if any, it is in very small spots and soon disappears. By the second or third day, however, ulcerations commence on the tonsils, and often extend rapidly in circumference and depth, with corresponding increase in the gravity of the general symptoms, until the condition of the patient becomes critical. It is only yesterday that I saw a case of this kind in consultation with Dr. T. A. Lilley, in the southwest part of the city. The patient was a boy, about seven years of age, who had been sick eight days, the throat was extensively ulcerated; the margins of the ulcers dark red; the voice husky; deglutition difficult; a brownish, sanious discharge from the nostrils; pulse quick, soft and weak; expression dull and typhoid, with the mind wandering or delirious a part of the time; yet no diphtheritic or white exhudation had been seen on the inflamed surface of the throat at any stage in the progress of the case.

In two cases belonging to this class, one a child, the other an adult, coming under my care, both tonsils were attacked with gangrene, accompanied by rapid prostration, and yet both ultimately recovered.

The case before us undoubtedly belongs to this class, the ulceration having commenced very early, preceded by only a very slight exhudation.

Regarding diphtheria in all its grades of severity as a general disease, caused by some specific influence or agent capable of disturbing the general properties of the tissues, altering the quality of the blood, and secondarily determining a specific inflammation of the fauces and glands of the neck, it has appeared to me of great practical importance to adopt such measures as were designed to counteract these effects at the earliest possible moment. As a general rule, the grade of morbid action induced, both general and local, is asthenic or depressing, as indicated by general impairment of tonicity and local ulcerations, followed, in the convalescence, by anæmia, muscular weakness, and sometimes paralysis. Consequently the treatment should be mainly antiseptic, corrective and tonic. As far as possible the patient should be supplied with an abundance of fresh, pure air, not overheated, but comfortable in temperature, and simple, easily assimilable food or nourishment, throughout the whole course of the disease, and until health is fully restored. Entire rest, mental and physical, should be enjoined during the progress of the disease, and over-exertion should be carefully avoided until the period of convalescence has fully passed by. In the first stage, which usually lasts from four to five days, I have found no remedies more beneficial than a combination of chlorate of potash, hydrochloric acid and belladonna, as in the following formulæ:

| | | |
|---|---|---|
| ℞ Potass. chlor. | 10 | grams. |
| Acid hydrochl. | 3 | " |
| Belladonnæ tinct. | 8 | " |
| Syrup. simpl. | 20 | " |
| Aquæ. | 115 | " |

Mix. Take one teaspoonful every two, three or four hours, according to the severity of the symptoms, for adults; and proportionately less, according to age, for children. In the milder class of cases this is all the medicine necessary during the first three or four days, unless the bowels should need moving, by a mild laxative or an enema. But in cases of medium or greater severity, I give, in addition, the benzoate of soda, dissolved in water, in the proportion of 10 grams of the first to 120 grams of the latter, of which an adult may take one teaspoonful between each of the doses of the chlorate solution, and children less in proportion. In cases of decided severity, in which the diphtheritic coating is accumulating rapidly, with corresponding increase of the glandular swellings in the neck, the application of dilute lactic acid in the form of spray to the fauces every three or four hours, and the application externally over the swollen glands, of a liniment containing chloroform, olive oil and oil of turpentine, will aid materially in checking the progress of the disease. In using the spray I put from three to five minims of the concentrated lactic acid to the ounce or 30 grams of water. The liniment consists of:

| | | |
|---|---|---|
| ℞ | Ol. oliv. | 100 grams. |
| | Ol. terebinth. | 15 " |
| | Chloroform. | 15 " |

Mix. Shake up at time of application. When the first stage has passed, as indicated by commencing disintegration of the membranous exudation, and perhaps ulceration, more abundant and offensive secretion, and weaker pulse, the use of the benzoate soda should be omitted or restricted to merely local use as a gargle, and proper doses of the tincture of chloride of iron and quinine substituted in its place. The regular administration of nourishment, consisting of milk, thin wheat-flour and milk gruel, beef tea, mutton broth, etc., should be more persistently attended to. In very bad cases, where enough of such nourishment cannot be swallowed to sustain the patient, the rectum should be made available by using nutritive enemas, and in the same cases much advantage can be obtained by applying cod-liver oil, holding in suspension a little quinine, over a large part of the cutaneous surface every four to six hours. During the last year I have seen two children in consultation, whose recovery appeared to be owing entirely to these latter means of support, for neither of them were able to take an ounce of anything by the stomach for more than a week. In the advanced stage of some cases accompanied by great nervous and vascular weakness, small doses of strychnine with nitric acid I have found more efficient than quinine. During the middle and latter stages of the disease I seldom make any local applications to the throat, except what the patient necessarily makes by swallowing in a dilute form such internal remedies as the tincture of chloride of iron, quinine and mineral acids. I do not believe that the disease originates locally from germs impinging on the mucous membrane of the the fauces and exciting inflammation, from whence the blood and system become inoculated, simply because in all the cases I have seen, symptoms of general disturbance have preceded the local appearances. After having tried, or seen tried by others, during a long series of years, almost every variety of treatment that has been suggested for the management of diphtheria, I am satisfied that a judicious use of the remedies just detailed to you, carefully adjusting each to the stage of the disease and condition of the patient, will result in more recoveries, and be followed by a less number of unpleasant sequelæ, than that of any other class of remedies. Even when the diphtheritic inflammation develops in the larynx, constituting diphtheritic croup, I have found more benefit from the frequent use of the lactic acid spray, one or two prompt emetics of the sub-sulphate of mercury in the beginning, and subsequently giving moderate doses of a solution of lactate of iron every one, two or three hours, according to the urgency of the symptoms, than from any other medical treatment. In these cases surgical treatment by tracheotomy may be available in some cases. In one of the cases I have alluded to, in which, instead of white exudation, gangrene showed itself almost from the beginning of the attack, accompanied by a weak and frequent pulse, much difficulty of deglutition, and general feeling of prostration, I prescribed at once salicylic acid and bicarbonate of soda dissolved in tincture of cinchona, to be given in doses of a teaspoonful, diluted with a tablespoonful of water, every two hours. The patient was a young man, and his progress was much more favorable than I had anticipated. The gangrene speedily ceased spreading, the sloughs separated in three or four days, and the resulting ulcers healed so rapidly that the patient was fairly convalescent in two weeks.

But the clinic hour having expired, I must not detain you longer at this time.

---

THE NICTITATING MEMBRANE IN MAN.—Dr. John Dell'Orto read before the New Orleans Medical and Surgical Association, October 9, 1880, the following:

I am glad that this subject offers me the opportunity to speak now of another peculiarity—this is a physiological one—that has been lately discovered on two negro women by Doctor Giacomini, the actual professor of anatomy of the University of Turin (Italy.)

At one of the meetings of the Academy of Medicine, of last year, he said that at the autopsy performed on these two persons (who were mother and daughter) he found in the semilunar folds of the conjunctiva, or third eye-lids, the same cartilage, common to birds and some small mammifera. It has been generally admitted, that this cartilage does not exist in apes and man, in whom the semi-lunar is constituted by a folding of the conjunctiva with the interposition of a composed connective tissue. But during the last few years Professor Giacomini had the occasion to observe with the microscope transversal sections of the semi-lunar folds of the eyes of one *ourang*—of two *cercopiteci* and of one *cinocephalus*—and he found in every one the same small quadrilateral cartilage that he found in these two women. These facts induced him to investigate on white people—in 320 eyes, from 160 white persons, he found the cartilage only in one instance.—*New Orleans Surgical and Medical Journal*, Dec., 1880.

